# Effective Assessment of Rheumatoid Arthritis Disease Activity and Outcomes Using Monocyte Chemotactic Protein-1 (MCP-1) and Disease Activity Score 28-MCP-1

**DOI:** 10.3390/ijms252111374

**Published:** 2024-10-23

**Authors:** Ping-Han Tsai, Lieh-Bang Liou

**Affiliations:** 1 Division of Rheumatology, Allergy, and Immunology, New Taipei Municipal Tucheng Hospital, New Taipei City 236, Taiwan; a9232@cgmh.org.tw; 2 Division of Rheumatology, Allergy, and Immunology, Chang Gung Memorial Hospital at Linkou, Taoyuan City 333, Taiwan; 3 School of Medicine, Chang Gung University College of Medicine, Taoyuan City 333, Taiwan

**Keywords:** DAS28-ESR, DAS28-MCP-1, HAQ-DI, MCP-1, residual joint swelling

## Abstract

The effectiveness of monocyte chemotactic protein-1 (MCP-1) and Disease Activity Score 28 (DAS28)-MCP-1 (DAS28-MCP-1) in assessing rheumatoid arthritis (RA) disease activity is unclear, although some studies have demonstrated their potential usefulness. The present study investigated relationships between MCP-1 and different DAS28 measures, the occurrence of residual swollen joints in different DAS28 remission statuses, changes in medication dosage in relation to the 2005 modified American Rheumatism Association and 2011 American College of Rheumatology/European League against Rheumatism (ACR/EULAR) remission definitions, and the correlations between different DAS28-related scores and Health Assessment Questionnaire Disability Index (HAQ-DI) scores in two RA patient cohorts. The results revealed that the MCP-1 level was correlated with five disease activity measures (DAS28-erythrocyte sedimentation rate [DAS28-ESR], DAS28-C-reactive protein [CRP], Simplified Disease Activity Index (SDAI), Clinical Disease Activity Index (CDAI), and DAS28-MCP-1) in multivariable regression analysis (all *p* < 0.05; ESR, CRP, and MCP-1 as independent variables). However, ESR was not significantly associated with SDAI and CDAI scores (*p =* 0.343 and 0.323, respectively). Residual swollen joints were more frequently observed in patients who met the DAS28-ESR remission criteria (<2.6) compared with those meeting the other four remission criteria, with a difference ranging from 71% to 94%. Among patients meeting the DAS28-ESR remission criteria (<2.6), medication changes (dose increase by ≥30% or new medications prescribed) were less frequent in those who also met the 2011 ACR/EULAR remission criteria than in those who did not meet them (*p* = 0.006). Moreover, the correlation coefficients for the relationship between DAS28-ESR and HAQ-DI scores were the lowest among the five disease activity measures. In conclusion, MCP-1 and DAS28-MCP-1 are effective in assessing RA disease activity, with less residual joint swelling and less frequent medication increases observed in the DAS28-MCP-1 remission < 2.2 subgroup.

## 1. Introduction

Remission is the primary goal in rheumatoid arthritis (RA) treatment, especially with the advent of biologic therapies. Several criteria exist to determine the feasibility of achieving RA remission, including the 28-joint Disease Activity Score (DAS28) index [1]. However, the 2011 American College of Rheumatology/European League against Rheumatism (ACR/EULAR) definition of RA remission [2] has been demonstrated to outperform the DAS28-erythrocyte sedimentation rate (DAS28-ESR) in terms of patient and physician global assessments as well as morning stiffness and functional status evaluations [3]. Furthermore, different remission criteria yield varied percentages of remission fulfillment, as demonstrated by the 1981 American Rheumatism Association (ARA) criteria [4] (6%), DAS28-ESR (24%), DAS28-C-reactive protein (DAS28-CRP, 100%), and the 2011 ACR/EULAR remission criteria (Boolean definition, 8%), with reported percentages of remission fulfillment ranging from 6% to 100% [5]. In particular, it had no agreement of kappa statistics between any two criteria [5].

Aletaha and Smolen argued that DAS28-based remission criteria should be abandoned [6] because residual joint swelling can occur even when the DAS28-ESR score is <2.0 [7], and residual tissue or molecular abnormalities can indicate the presence of active disease [8]. Although numerous studies have reported that DAS28-based remission criteria are unsuitable for clinical practice, we propose a clinically valuable alternative, namely the DAS28 score supplemented by monocyte chemotactic protein-1 (MCP-1) results (DAS28-MCP-1) [9,10]. MCP-1 is a key chemokine produced by activated monocytes and fibroblasts at the sites of inflammation [11] and regulates the migration and infiltration of monocytes and macrophages. It is primarily secreted by synovial fibroblasts in response to inflammatory cytokines such as interleukin (IL)-1, tumor necrosis factor-α, and interferon-γ [12]. In addition to recruiting monocytes, MCP-1 activates both monocytes and macrophages, prompting them to release IL-1 and IL-6 [13]. This process further promotes the production of chemokines and pro-inflammatory cytokines through autocrine and paracrine feedback loops [13]. Abnormal expression of MCP-1 can also drive the transformation of monocytes into macrophages within the knee joint capsule, stimulate osteoclasts for bone resorption, induce inflammation, ultimately leading to joint destruction [14,15], and play a crucial role in rheumatoid arthritis [16]. A study reported that an MCP-1 antagonist reduced or prevented arthritis in MRL-lpr mice, indicating that MCP-1 is likely involved in arthritic inflammation [17]. We demonstrated the high criterion validity of DAS28-MCP-1 by identifying strong intercorrelations and time-change correlations among various disease activity scores as well as the favorable limits of agreement by using Bland–Altman plots [10]. Additionally, we established the high construct validity of DAS28-MCP-1 by identifying similar correlations between various disease activity scores and Health Assessment Questionnaire Disability Index (HAQ-DI) scores [10]. Our results revealed that the DAS28-ESR score was not consistent with the 2011 ACR/EULAR remission criteria (both the Boolean definition and the Simplified Disease Activity Index (SDAI) threshold of ≤3.3) [10]. By contrast, DAS28-MCP-1 favorably aligned with both definitions of the 2011 ACR/EULAR remission criteria [10].

This study’s objective is to determine whether residual joint swelling is more prevalent in patients with DAS28-ESR scores below 2.6 and whether adjustments in medication (either adding new medications or modifying doses) were made for patients meeting two DAS28-based remission criteria ([2,18]—the 2005 modified ARA remission); hence, we reanalyzed data from the 2013 and 2020 RA patient cohorts [9,10]. Moreover, another study objective is that we re-examined these cohorts to evaluate whether ESR, CRP, and MCP-1 levels are associated with different DAS28-based scores and with the tender joint count (TJC) and swollen joint count (SJC) [9,10].

## 2. Results

Table 1 summarizes the characteristics of the 2013 and 2020 RA patient cohorts. The 2013 cohort comprised 111 patients and the 2020 cohort comprised 178 patients. Both cohorts were predominantly female and exhibited similar disease activity levels, ranging from remission/low to high. The levels of ESR, CRP, and MCP-1 in both cohorts were either normal or above normal values (that is, displayed whole ranges of biomarker values). The 2013 cohort included only data from Month 0, with a limited number of patients followed up in subsequent months; hence, the follow-up visits are not calculated. In contrast, the 2020 cohort had follow-up visits at Months 3, 6, 9, and 12, achieving 93.8% of expected patient visits (835 visits out of 890 visits).

### 2.1. Association of Biomarkers with DAS28-Related Scores, Tender Joint Count, and Swollen Joint Count

We reanalyzed the 2013 RA patient cohort [9] by using multivariable regression analysis and determined that plasma MCP-1 and blood ESR levels were significantly correlated with DAS28-ESR scores. However, serum CRP levels were not significantly correlated with DAS28-ESR scores (Table 2). Furthermore, plasma MCP-1 and serum CRP levels were significantly correlated with DAS28-CRP scores. However, blood ESR levels were not significantly correlated with DAS28-CRP scores (Table 2). Only plasma MCP-1 levels were significantly correlated with DAS28-MCP-1 scores. No significant correlations were noted between ESR and CRP and DAS28-MCP-1 scores (Table 2). These results of the multivariable regression analysis suggest that plasma MCP-1 alone is a reliable indicator of RA disease activity measures, in contrast to ESR and CRP.

In our reanalysis of the 2020 RA patient cohort [10], the results of the multivariable regression analysis revealed that serum MCP-1 and CRP, and blood ESR levels, were significantly correlated with DAS28-ESR, DAS28-CRP, and DAS28-MCP-1 scores (Table 3). Furthermore, serum MCP-1 and CRP but not blood ESR levels were significantly correlated with the SDAI and Clinical Disease Activity Index (CDAI; Table 3). These results suggest that serum MCP-1 and CRP levels are closely associated with five DAS28-related scores. However, blood ESR levels were only partially associated with these five DAS28-related scores (Table 3).

Table 4 illustrates the correlation of ESR, CRP, and MCP-1 with the TJC and SJC in two cohorts (2013 and 2020). MCP-1 consistently had significant associations with both the TJC and SJC across both cohorts, while ESR and CRP display variable significant associations. Notably, CRP had significant associations with both the TJC and SJC in the 2020 cohort, while ESR had a significant association only with the SJC in the 2013 cohort. MCP-1’s consistent good performance suggests that it may be a more reliable marker for joint inflammation compared with ESR and CRP.

### 2.2. Expression of Joint Swelling in Different DAS28-Based Remission Criteria

The committee that established the 2011 ACR/EULAR remission criteria for RA reported that “10% of patients with a DAS28 score of <2.6 had ≥4 swollen joints, and one patient had >20 swollen joints” [19]. Similarly, our reanalysis of the same dataset indicated that 5.2% of the included patients with RA and a DAS28-ESR score of <2.6 had ≥4 swollen joints. One of these patients even had 10 swollen joints [10]. However, in the same cohort [10], none of the patients with RA and a DAS28-CRP score of <2.5 or a DAS28-MCP-1 score of <2.2 had ≥4 swollen joints (Table 5). Although the committee’s editorial [19] used data from “a bank of RA trial data”, we analyzed data from a real-world outpatient sample [10].

Residual swollen joints were more frequently reported (a difference of 71–94%) during visits where patients achieved remission in accordance with DAS28-ESR criteria (<2.6) compared with those where patients achieved remission in accordance with the other four disease activity scores (Table 5). This trend was also observed among patients with RA who achieved remission in accordance with disease activity scores but still had ≥2 swollen joints (a difference of 75–91%; Table 5).

### 2.3. Fulfillment of Two Remission Definitions by Different DAS28-Based Remission Criteria, Together with Medication Changes

The percentage of patients who required increased medication doses or additional medications (any disease-modifying antirheumatic drugs) was higher but nonsignificant among those who met the DAS28-ESR remission criteria but did not meet the 2005 modified ARA definition of remission than among those who met the 2005 modified ARA definition of remission, which is the most stringent remission standard defined by the absence of tender and swollen joints [18]. As presented in Table 6, we examined medication changes by first evaluating patients with RA who achieved remission in accordance with various disease activity scores at Months 0, 3, or 6 (baseline). We then compared their medication use 6 months later with that at baseline to determine whether their medication dose had increased by ≥30% or whether new medications had been prescribed. Similarly, the percentage of patients who required increased medication doses or new medications did not significantly differ between those who did not achieve remission per the 2005 modified ARA definition of remission and those who did among patients who met the DAS28-CRP, SDAI, CDAI, or DAS28-MCP-1 remission criteria (Table 6). Visits with a medication dose increase were listed in a decreasing order: Methotrexate > Sulfasalazine > Prednisolone > Hydroxychloroquine > Leflunomide > Azathioprine > Cyclosporine. And those visits with newly added medications were also listed in a decreasing order: Hydroxychloroquine > Methotrexate > Prednisolone > Golimumab > Sulfasalazine = Leflunomide > Azathioprine > Cyclosporine.

Among patients who met the DAS28-ESR remission criteria, significant differences in medication changes were observed between those who also met the 2011 ACR/EULAR remission criteria and those who did not meet them (Table 6). By contrast, among patients who met the DAS28-CRP, SDAI, CDAI, or DAS28-MCP-1 remission criteria, no significant medication change was noted between those who met the 2011 ACR/EULAR remission criteria and those who did not meet them (Table 6).

Finally, the DAS28-MCP-1, DAS28-CRP, SDAI, and CDAI criteria significantly outperformed the DAS28-ESR criteria, producing results that aligned well with the 2011 ACR/EULAR remission criteria (Table 6). Furthermore, compared with the DAS28-ESR, SDAI, and CDAI remission criteria, the DAS28-MCP-1 remission criteria aligned significantly better with the 2005 modified ARA remission criteria (Table 6).

## 3. Discussion

In RA treatment, remission is the primary goal of a treat-to-target strategy [20,21], and effectively managing RA can considerably improve a patient’s health-related quality of life (QoL) [22,23]. A study reported that remission defined by the DAS28-ESR, SDAI, and Boolean criteria at baseline was significantly associated with more favorable outcomes on the Health Assessment Questionnaire (HAQ), Short Form 36, EuroQol, and Functional Assessment of Chronic Illness Therapy [24]. Among these, Boolean-defined remission had the most substantial positive effect on health-related QoL, whereas DAS28-ESR-defined remission had the least substantial effect. Moreover, of the 17 remission definitions studied, the highest and lowest proportions of patients with an HAQ score of ≤0.5 were found among those who achieved Boolean-defined remission (88.8%) and DAS28-ESR-defined remission (67.5%), respectively [25]. The findings of these studies indicate that DAS28-ESR-defined remissions are inadequate for determining the functional status of patients with RA.

To evaluate the correlations between various disease activity scores and HAQ-DI results throughout our 1-year follow-up period (with assessments conducted every 3 months), we reanalyzed the same dataset [10]. We compared the correlation coefficients for the relationship between DAS28-ESR and HAQ-DI scores with those for the relationships of DAS28-CRP, SDAI, CDAI, and DAS28-MCP-1 scores with HAQ-DI scores. The findings revealed that the correlations of DAS28-CRP, SDAI, and CDAI scores with HAQ-DI scores were significantly stronger than the correlation of DAS28-ESR scores with HAQ-DI scores at Months 0, 6, 9, and 12 (Table 7). No significant differences were noted at Month 3. Moreover, at Month 6, no significant difference was observed between the correlations of DAS28-ESR and DAS28-CRP scores with HAQ-DI scores (Table 7). Although the correlations of DAS28-MCP-1 with HAQ-DI scores did not differ from those of DAS28-ESR with HAQ-DI scores at any time point, the correlations of DAS28-MCP-1 with HAQ-DI scores at Months 3, 6, and 9 were also not different from those of DAS28-CRP, SDAI, and CDAI scores with HAQ-DI scores (all *p* > 0.05), except for the comparison of the correlation between DAS28-MCP-1 with HAQ-DI scores and SDAI with HAQ-DI scores at Month 9 (*p* = 0.01).

The limitation of our study is that we have not yet established the criteria for assessing treatment response in RA patients using DAS28-MCP-1. Additionally, the utility of DAS28-MCP-1 in evaluating bone changes through X-ray examination in RA patients has not been confirmed. Lastly, further studies are needed to validate the effectiveness of DAS28-MCP-1 in assessing RA disease activity across different racial populations.

In summary, the correlation coefficients for the relationship between DAS28-ESR and HAQ-DI scores were the lowest among all the disease activity measures tested.

Whether ESR, CRP, and MCP-1 (as independent variables) correlate with the tender joint count (TJC) and swollen joint count (SJC) (as dependent variables) has been examined earlier (the 2013 cohort) but reanalyzed and displayed in Table 4 [9]. The same was performed for the 2020 cohort (Table 4). In both cohorts, it demonstrated that MCP-1 correlated significantly with the TJC and SJC, in contrast to only partly of ESR and CRP (Table 4). Therefore, MCP-1 is the best among three biomarkers (ESR, CRP, and MCP-1) to correlate with the TJC and SJC.

The superiority of DAS28-MCP-1 over DAS28-ESR in terms of the fulfillment of the 2005 modified ARA definition of remission and the 2011 ACR/EULAR remission criteria is demonstrated not only in Table 6 [10], but also in another previous publication [26]. Moreover, DAS28-MCP-1 outperformed the SDAI and CDAI remission criteria in the fulfillment of the 2005 modified ARA remission criteria (Table 6).

## 4. Materials and Methods

### 4.1. Participants and Study Design

The Institutional Review Board of Chang Gung Memorial Hospital, Taoyuan City, Taiwan, approved the study protocols for the 2013 [9] and 2020 [10] studies. Both studies consecutively enrolled patients who met the 1987 American College of Rheumatology criteria for RA during 2006–2008 and during 2013–2016, respectively. We determined the sample sizes for the 2013 and 2020 cohorts based on the research and experimental designs of van der Heijde, D. M. et al. [27] and Prevoo, M. L. et al. [28] for the former, and Aletaha, D. et al. [29] and Mäkinen, H. et al. [18] for the latter. The study included 111 patients in the 2013 cohort and 178 patients in the 2020 cohort (with total 835 visits), all of whom provided written informed consent. In the 2013 cohort, patients with infections or acute cardiopulmonary compromise were excluded. In the 2020 cohort, patients with at least one tender or swollen joint—indicating a higher potential for changes in DAS28-ESR scores—were selected as candidates after giving written informed consent. Follow-up visits occurred every 3 months over a 12-month period, with five visits in total. Each patient had 20 mL of blood collected for testing of ESR, CRP, and MCP-1 (R&D Systems, Minneapolis, MN, USA), along with data collection on TJC, SJC, patient global assessment (PGA), evaluator global assessment (EGA), HAQ-DI scores, and the duration of morning stiffness (in minutes). The central laboratory of Chang Gung Memorial Hospital systems has designated normal values of ESR as <15 mm/h and CRP as <5.0 mg/L. The normal values of MCP-1 in healthy controls examined by us earlier are 70.25 ± 16.70 pg/mL (range 0.00–473.34) [9]. The limit of detection for CRP is 0.20 mg/L for the 2020 cohort (measured by turbidimetric immunoassay in serum), that for CRP is 3.00 mg/L for the 2013 cohort (measured by nephelometry in serum), and that for MCP-1 is 15.6 pg/mL (measured by ELISA assay in plasma [the 2013 cohort] and serum [the 2020 cohort]).

### 4.2. Calculation of DAS28 Scores

DAS28 scores were calculated using the following formulas: $DAS28-ESR\;score=[0.56\times \sqrt{TJC]}+[0.28\times \sqrt{SJC}]+[0.70\times \ln(ESR)]+[0.014\times PGA$ (VAS; in mm)]; $DAS28-CRP\;score=[0.56\times \sqrt{TJC]}+[0.28\times \sqrt{SJC]}+[0.36\times \ln\left(CRP:in\;mg/L\right)+1]+[0.014\times PGA\left(VAS;\;in\;mm\right)]+0.96$; $SDAI\;score=TJC+SJC+PGA\left(VAS;in\;cm\right)+EGA(VAS;in\;cm)+CRP(in\;mg/dL)$; and $DAS28-MCP-1\;score=\left[0.56\times \sqrt{TJC}\right]+\left[0.28\times \sqrt{SJC}\right]+[0.39\times \ln\left(MCP-1\right)(in\;pg/mL)+[0.014\times PGA$ $\left(VAS:in\;mm\right)]$ [10].

CDAI scores were calculated as follows: $CDAIscore=TJC+SJC+PGA\left(VAS;incm\right)+EGA(VAS;incm)$ [29]. VAS represents visual analog scale.

### 4.3. Statistical Analysis

Statistical analyses were conducted using SPSS version 16.0 (IBM, SPSS Inc., Chicago, IL, USA). The one-sample Kolmogorov–Smirnov Z test was used to assess data normality. Multivariable regression analysis was performed to evaluate correlations between biomarkers and disease activity measures in the two RA patient cohorts. Odds ratios were calculated to compare residual SJCs or remission rates across various Disease Activity Score-based remission statuses among patients with RA. Comparisons between correlation coefficients were performed using the online calculator for testing correlations [30].

## 5. Conclusions

The serum MCP-1 level is the most favored biomarker for evaluating RA disease activity measures because it is correlated with all five scoring formulas (DAS28-ESR, DAS28-CRP, SDAI, CDAI, and DAS28-MCP-1), unlike the ESR and CRP levels. Moreover, the serum MCP-1 level correlates preeminently with the TJC and SJC, compared with the correlations of the TJC and SJC with ESR and CRP. Also, “the cost of the MCP-1 tests using the ELISA assay is 0.58 times lower than that of the CRP test performed by” turbidimetric immunoassay, “which is the current method used in our hospital system across four hospital clusters” (10 separate branches) in Taiwan [10]. This cost-effectiveness makes MCP-1 a viable alternative to CRP, a traditional biomarker, for evaluating the disease activity of rheumatoid arthritis in clinical settings.

The results obtained using the proposed DAS28-MCP-1 formula aligned well with other commonly used disease activity measures. In particular, DAS28-MCP-1 scores were consistent with the 2011 ACR/EULAR remission criteria, which are compatible with the SDAI, CDAI, and DAS28-CRP results. Thus, DAS28-MCP-1 significantly outperformed DAS28-ESR in this regard. Both DAS28-MCP-1 and DAS28-CRP scores aligned well with the 2005 modified ARA definition of remission, which is considered the most stringent definition of RA remission, significantly outperforming the DAS28-ESR, SDAI, and CDAI. Additionally, we previously published that DAS28-MCP-1 shows significant differences in relation to specific immunoregulatory cytokines and ultrasound grades compared to DAS28-ESR, DAS28-CRP, and SDAI, suggesting that the underlying mechanisms behind evaluation by these formulas may differ [26]. The mechanism underlying this difference requires further investigation.

Patients who achieved remission per the DAS28-ESR remission criteria (<2.6) had a greater number of residual swollen joints and made more visits with residual swollen joints and ≥2 swollen joints compared with those who achieved remission in accordance with the other four types of disease activity scores.

The correlation coefficients for the relationship between DAS28-ESR and HAQ-DI scores were the lowest among all disease activity measures for most of the 12-month period. Importantly, all messages secured in this manuscript are complementary to the known information published in refs. [9,10] to confer a complete development for a new laboratory biomarker of MCP-1 and a new DAS28 evaluation formula of DAS28-MCP-1 in assessing the disease activity of patients with rheumatoid arthritis.

Future studies are suggested to involve establishing the criteria for assessing treatment response in RA patients using DAS28-MCP-1 and confirming X-ray changes related to changes in DAS28-MCP-1 scores.

## Figures and Tables

**Table 1:** Demographic, clinical, and laboratory data of patients with RA in two cohorts.

	The 2013 Cohort		The 2020 Cohort	
Variables	Mean ± S.D. or Median	Range or Inter-Quartile Ranges (25th, 75th)	Mean ± S.D. or Median	Range or Inter-Quartile Ranges (25th, 75th)
Number of patients	111	F:M = 4.05:1 (89:22)	178	F:M = 3.94:1 (142:36)
Total visits in 12 months	-	-	835	-
ESR (mm/h)	41.29 ± 28.56	4–128	17.50	(9.75–36.00)
CRP (mg/L)	10.10	(3.30–28.60)	3.90	(0.24–133.15)
MCP-1 (pg/mL)	58.63	(31.5–160.35)	101.8	(73.6–140.1)
Tender joint count	6.7 ± 5.5	0–28	5.0	(2.0, 9.3)
Swollen joint count	4.0	(1.0, 7.0)	2.0	(1.0, 5.3)
DAS28-ESR scores	5.2 ± 1.6	1.6–8.6	4.3 ± 1.4	0.8–7.3
DAS28-CRP scores	4.7 ± 1.6	1.5–7.9	4.8 ± 1.3	2.5–8.3
SDAI	-	-	15.3	(9.1, 23.7)
CDAI	-	-	10.0	(5.0, 17.0)
DAS28-MCP-1 scores	4.9 ± 1.6	2.4–9.9	4.2 ± 1.0	2.2–6.8

RA: rheumatoid arthritis; DAS28: Disease Activity Score of 28 joints; SDAI: Simplified Disease Activity Index; CDAI: Clinical Disease Activity Index. A majority portion of Table 1 was taken from refs. [9,10]. Normal values: ESR < 15 mm/h; CRP < 5.0 mg/L; monocyte chemotactic protein-1 (MCP-1): 70.25 ± 16.70 pg/mL.

**Table 2:** Multivariable analysis ^#^ of correlations between biomarkers and disease activity measures for the RA patient cohort 2013 *.

Dependent Variables	Independent Variables	t-Values	*p*-Values
DAS28-ESR scores			
	ESR	5.640	<0.001
	CRP	0.883	0.407
	MCP-1	2.987	0.003
DAS28-CRP scores			
	ESR	0.587	0.559
	CRP	4.390	<0.001
	MCP-1	2.513	0.013
DAS28-MCP-1 scores			
	ESR	1.953	0.053
	CRP	1.457	0.148
	MCP-1	0.618	<0.001

^#^ Regression analysis was performed with each dependent variable and three independent variables to obtain t- and *p*-values. * Data from the same patient cohort as in ref. [9] (n = 111 patients). RA: rheumatoid arthritis; DAS28: Disease Activity Score 28; MCP-1: monocyte chemotactic protein-1. *p*-values of <0.05 were considered statistically significant.

**Table 3:** Multivariable analysis ^#^ of correlations between biomarkers and disease activity measures for the RA patient cohort 2020 *.

Dependent Variables	Independent Variables	t-Values	*p*-Values
DAS28-ESR scores			
	ESR	13.642	<0.001
	CRP	2.650	0.008
	MCP-1	2.343	0.019
DAS28-CRP scores			
	ESR	3.314	0.001
	CRP	9.842	<0.001
	MCP-1	2.283	0.023
SDAI			
	ESR	0.949	0.343
	CRP	6.936	<0.001
	MCP-1	2.441	0.015
CDAI			
	ESR	0.989	0.323
	CRP	3.839	<0.001
	MCP-1	2.465	0.014
DAS28-MCP-1 scores			
	ESR	2.001	0.046
	CRP	3.684	<0.001
	MCP-1	7.196	<0.001

^#^ Regression analysis was performed with each dependent variable and three independent variables to obtain t- and *p*-values. * Data from the same patient cohort as in ref. [10] (n = 835 visits). RA: rheumatoid arthritis; DAS28: Disease Activity Score 28; MCP-1: monocyte chemotactic protein-1. *p*-values of <0.05 were considered statistically significant.

**Table 4:** Multivariable analysis ^#^ of correlations between tender joint count or swollen joint count and three biomarkers *.

Dependent Variables	Independent Variables	t-Values	*p*-Values
TJC (the 2013 cohort)			
	ESR	1.226	0.223
	CRP	0.610	0.543
	MCP-1	2.987	0.003
TJC (the 2020 cohort)			
	ESR	0.605	0.545
	CRP	3.256	0.001
	MCP-1	2.161	0.031
SJC (the 2013 cohort)			
	ESR	2.641	0.010
	CRP	1.929	0.056
	MCP-1	3.423	0.001
SJC (the 2020 cohort)			
	ESR	0.829	0.407
	CRP	6.718	<0.001
	MCP-1	4.322	<0.001

^#^ Regression analysis was performed with each dependent variable and three independent variables to obtain t- and *p*-values. * Data from the same patient cohort as in ref. [9] (n = 111 patients) and in ref. [10] (n = 835 visits). TJC: tender joint count; SJC: swollen joint count; MCP-1: monocyte chemotactic protein-1. *p*-values of <0.05 were considered statistically significant.

**Table 5:** Associations of residual swollen joint count with various Disease Activity Score-based remission statuses among patients with rheumatoid arthritis.

	The Remaining SJC Based on Individual Patients	The Remaining SJC Based on Patient Visits	The Remaining SJC ≧ 2 Based on Patient Visits
DAS28-ESR (<2.6)	38.7/26 = 1.49 ± 1.92 (reference)	62/38 = 1.63 ± 1.57 (reference)	36/12 = 3.00 ± 2.30 (reference)
DAS28-CRP (<2.5)	2.5/2 = 1.25 ± 1.06 (*p* = 0.806)	3/2= 1.50 ± 0.71 (*p* = 0.841)	2/1 = 2.00 (*p* = 0.684)
SDAI (≦3.3)	6.3/9 = 0.71 ± 0.56 (*p* = 0.07)	11/9 = 1.22 ± 0.44 (*p* = 0.170)	4/2 = 2.00 ± 0.00 (*p* = 0.160)
CDAI (≦2.8)	7.8/11 = 0.72 ± 0.51 (*p* = 0.07)	14/11 = 1.27 ± 0.47 (*p* = 0.223)	6/3 = 2.00 ± 0.00 (*p* = 0.160)
DAS28-MCP-1 (<2.2)	4.5/4 = 1.13 ± 0.63 (*p* = 0.466)	6/4 = 1.50 ± 0.58 (*p* = 0.740)	4/2 = 2.00 ± 0.00 (*p* = 0.160)

The results in Table 3 were reanalyzed from the same dataset used in ref. [10]. Numerators represent the total number of swollen joints, and denominators indicate the number of patients or visits with joint swelling or ≥2 swollen joints. Parentheses show *p*-values for comparisons between the given group and the reference group by using odds ratios. Abbreviations: DAS28-ESR, Disease Activity Score 28–erythrocyte sedimentation rate; DAS28-CRP, Disease Activity Score 28–C-reactive protein; SDAI, Simplified Disease Activity Index; CDAI, Clinical Disease Activity Index; DAS28-MCP-1, Disease Activity Score 28–monocyte chemotactic protein-1.

**Table 6:** Associations of remission rates with various Disease Activity Score-based statuses and the need for increased drug dosage or additional drugs among patients with rheumatoid arthritis.

	Remission Status	Odds Ratio ^a^	The Remission Group (%): Dose Change or a New Drug	The Non-Remission Group (%): Dose Change or a New Drug
Fulfilled the 2005 modified ARA remission ^b^				
DAS28-ESR (<2.6)	36.99% (64/173)	Reference	2/22 = 9.09%	17/56 = 30.36%
DAS28-CRP (<2.5)	70.00% (49/70)	3.97	2/18 = 11.11%	2/7 = 28.57%
SDAI (≦3.3)	54.10% (66/122)	2.01	2/22 = 9.09%	2/24 = 8.33%
CDAI (≦2.8)	53.33% (64/120)	1.44	2/22 = 9.09%	2/23 = 8.70%
DAS28-MCP-1 (<2.2)	75.61% (61/82)	5.28	2/21= 9.52%	0/5 = 0.00%
Fulfilled the 2011 ACR/EULAR remission ^c^				
DAS28-ESR (<2.6)	49.13% (85/173)	Reference	2/30 = 6.67% *	17/48 = 35.42% *
DAS28-CRP (<2.5)	82.86% (58/70)	5.00	2/21 = 9.52%	2/4 = 50.00%
SDAI (≦3.3)	81.97% (100/122)	4.71	4/38 = 10.52%	0/8 = 0.00%
CDAI (≦2.8)	80.83% (97/120)	1.65	4/38 = 10.52%	0/7 = 0.00%
DAS28-MCP-1 (<2.2)	81.71% (67/82)	4.62	2/23 = 8.70%	0/3 = 0.00%

Abbreviations are the same as those used in Table 1. ^a^ *p* < 0.01 for all odds ratios versus the reference in each category, except for the CDAI versus DAS28-ESR in relation to the 2005 modified American Rheumatism Association (ARA) definition of remission (*p* = 0.085). ^b^ 2005 modified ARA definition of remission [18]; ^c^ 2011 American College of Rheumatology/European League Against Rheumatism remission criteria [2]; parenthesized numbers indicate the number of visits. * *p* = 0.006 by Fisher’s exact test. *p* > 0.05 for all other comparisons between the remission and non-remission groups. Part of the data (remission status and odds ratio) were taken from ref. [10].

**Table 7:** Comparisons of correlation coefficients for relationships between different DAS28-related disease activity measures and HAQ-DI scores in patients with RA.

Measures	M0 (n = 178) Correlation Coefficients (*p*-Values against the Reference)	M3 (n = 163) Correlation Coefficients (*p*-Values against the Reference)	M6 (n = 163) Correlation Coefficients (*p*-Values against the Reference)	M9 (n = 162) Correlation Coefficients (*p*-Values against the Reference)	M12 (n = 169) Correlation Coefficients (*p*-Values against the Reference)
DAS28-ESR	0.553 (reference)	0.486 (reference)	0.607 (reference)	0.603 (reference)	0.524 (reference)
DAS28-CRP	0.626 (0.002) *	0.526 (0.094)	0.627 (0.216)	0.690 (0.001) *	0.593 (0.009) *
SDAI	0.650 (<0.001) *	0.541 (0.071)	0.686 (0.015) *	0.697 (0.003) *	0.633 (0.001) *
CDAI	0.639 (0.003) *	0.550 (0.059)	0.681 (0.029) *	0.677 (0.024) *	0.634 (0.002) *
DAS28-MCP-1	0.564 (0.372)	0.522 (0.172)	0.650 (0.086)	0.647 (0.112)	0.583 (0.059)

DAS28: Disease Activity Score 28; HAQ-DI: Health Assessment Questionnaire Disability Index; M0, M3, M6, M9, and M12: Month 0, Month 3, Month 6, Month 9, and Month 12, respectively. * Indicates comparisons with the reference at *p* < 0.05.

## Data Availability

The data that support the findings of this study are displayed in the article. Others are available from the corresponding author upon reasonable request.
